# Flexure Behaviors of ABS-Based Composites Containing Carbon and Kevlar Fibers by Material Extrusion 3D Printing

**DOI:** 10.3390/polym11111878

**Published:** 2019-11-13

**Authors:** Kui Wang, Shixian Li, Yanni Rao, Yiyun Wu, Yong Peng, Song Yao, Honghao Zhang, Said Ahzi

**Affiliations:** 1 Key Laboratory of Traffic Safety on Track of Ministry of Education, School of Traffic & Transportation Engineering, Central South University, Changsha 410075, China; kui.wang@csu.edu.cn (K.W.); shixian.li@csu.edu.cn (S.L.); raoyn1@csu.edu.cn (Y.R.); yiyun_wu@csu.edu.cn (Y.W.); song_yao@csu.edu.cn (S.Y.); 2 Joint International Research Laboratory of Key Technology for Rail Traffic Safety, Central South University, Changsha 410075, China; 3 National & Local Joint Engineering Research Center of Safety Technology for Rail Vehicle, Central South University, Changsha 410075, China; 4 ICUBE Laboratory—CNRS, University of Strasbourg, 67000 Strasbourg, France; sahzi@hbku.edu.qa; 5 Qatar Environment and Energy Research Institute (QEERI), Hamad bin Khalifa University (HBKU), Qatar Foundation, Doha, Qatar

**Keywords:** material extrusion 3D printing, short fiber reinforcements, flexural behavior, raster orientation, build direction

## Abstract

Short-fiber-reinforced thermoplastics are popular for improving the mechanical properties exhibited by pristine thermoplastic materials. Due to the inherent conflict between strength and ductility, there are only a few successful cases of simultaneous enhancement of these two properties in polymer composite components. The objective of this work was to explore the feasibility of simultaneous enhancement of strength and ductility in ABS-based composites with short-carbon and Kevlar fiber reinforcement by material extrusion 3D printing (ME3DP). Microstructure characterization and measurement of thermal and mechanical properties were conducted to evaluate the fiber-reinforced ABS. The influence of printing raster orientation and build direction on the mechanical properties of material extrusion of 3D-printed composites was analyzed. Experimental results demonstrated that the reinforcement of the ABS-based composites by short-carbon and Kevlar fibers under optimized 3D-printing conditions led to balanced flexural strength and ductility. The ABS-based composites with a raster orientation of ±45° and side build direction presented the highest flexural behaviors among the samples in the current study. The main reason was attributed to the printed contour layers and the irregular zigzag paths, which could delay the initiation and propagation of microcracks.

## 1. Introduction

The additive manufacturing (AM) technique is one of the most versatile and revolutionary methods for creating three-dimensional (3D) objects with complex structural components and diverse properties [1,2]. AM technologies consist of various methods, such as stereolithography apparatus (SLA) techniques [3], polyjet printing [4], fused deposition modeling (FDM) [5,6], selective laser sintering (SLS) [7], laser engineered net shaping (LENS) [8], and laminated object manufacturing (LOM) [9]. Material extrusion 3D printing based on fused deposition modeling (FDM) technology is of particular interest due to its affordability, minimal material wastes, environmental friendliness, and low learning curve [10,11].

Commercial thermoplastics, such as acrylonitrile butadiene styrene (ABS) [12], polylactic acid (PLA) [13,14], and polyamide (PA) [15], are widely used in material extrusion 3D printing. In most cases, the printed components using only pristine thermoplastics are good for prototype model demonstrations and are not strong enough for structural purposes [16]. In the plastics industry, short fibers, including carbon fibers (CFs), Kevlar fibers (KFs), and glass fibers (GFs), are typical reinforcements used to improve the mechanical behavior of thermoplastic-based composites using material extrusion 3D printing. Tekinalp et al. [17] studied the mechanical performances of ABS composites filled with different contents of CFs. They confirmed that short-CF-reinforced 3D-printed composites exhibited improved tensile strength that increased with increasing CF content. This was attributed to the addition of more rigid CF fiber inclusions. Ning et al. [18] evaluated the effect of CF weight ratios on the tensile strength and ductility of ABS composites fabricated by material extrusion 3D printing. They concluded that the tensile strength and Young’s modulus of the composites increased with increasing content of the CFs. The short-CF-reinforced ABS samples could support an increased load during the bending process. However, their ductility decreased with increasing content of CFs from 0 wt % to 10 wt %. Ferreira et al. [19] studied the mechanical behavior of PLA and PLA-based laminated materials reinforced with short CFs produced by material extrusion 3D printing. The tensile stiffness of the PLA composites increased with an increasing amount of the CFs. It should be noted that the short CFs in their composites were mostly aligned with the tensile direction. As in most studies, an increase in composite strength resulted in a decrease in the elongation of PLA/CF composites due to the presence of the CFs, indicating that the brittleness of reinforced composites increases with the addition of short CFs. Similarly, the mechanical properties of material extrusion 3D-printed continuous KF-reinforced composites were investigated by tensile and flexural tests by Dickson et al. [20]. The addition of KFs increased the elastic modulus and toughness of the PA-based composites, indicating the toughening effects provided by the KFs. In the literature, studies with improvements in both the mechanical strength and ductility for materials via 3D printing are limited. In most modified composites, the strength is enhanced, while the elongation is sacrificed. However, there are many applications that require both sufficient rigidity and ductility, such as for energy absorption purposes [21,22].

Mechanical behaviors of materials processed by material extrusion 3D-printing technologies highly depend on the printing parameters, such as printing temperature, raster orientation, and extrusion feeding rate [23,24]. This was revealed in the study by Ahn et al. [5], where the mechanical properties of ABS parts fabricated by material extrusion 3D printing were measured. They found that the mechanical properties of the materials were affected by the printed raster orientations. Specifically, the tensile strengths of the composites with a crisscross raster printing angle of [−45°/45°] were lower than those for the materials with a cross-raster printing angle of [0°/90°]. Similarly, Ziemian et al. [25] analyzed the effects of raster orientations on the flexural properties of ABS components fabricated by material extrusion 3D printing. The results indicated that the highest strength was obtained when the raster orientation was at 0°. The raster orientation of 0° offered a high resistance to bending because of its long, effective raster lengths. 

In this work, specimens of pristine ABS and ABS-based composites filled with CFs and/or KFs were prepared by material extrusion 3D printing with different raster orientations and different building directions. The mechanical properties of the materials were evaluated by means of flexural characterization. The microstructure was characterized using scanning electron microscopy (SEM) before and after flexural testing. The deformation and failure mechanisms of the materials were investigated by fractography. 

## 2. Experimental Methodology

### 2.1. Materials and Processing

In this study, ABS, ABS/CF, ABS/KF, and ABS/CF/KF filament spools were purchased from Nanovia (Saint Paul, France). The materials were supplied in the form of filament tows with a standard diameter of 1.75 mm. Table 1 presents mean length, mono diameters, and population of fibers acquired from the datasheet of supplier. Before processing, the filaments were kept in a drying oven to reduce the relative humidity.

**Figure 1:**
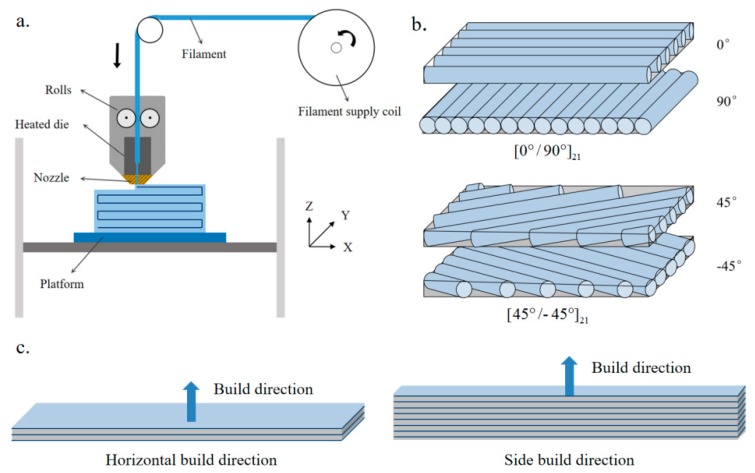
Schematic representation of (**a**) material extrusion 3D-printing process, (**b**) printing process with raster orientations along [0°/90°] and [±45°], and (**c)** specimen with horizontal and side build directions.

The filament tows were utilized to fabricate ABS and ABS composite samples using a desktop 3D printer (Raise3D N2 Plus, Raise3D INC., Costa Mesa, CA, USA). A diagram of the printing process is shown in Figure 1. The printing process consisted of melting and extrusion. During melting, the filaments were rolled into the heated die. When the extrusion head was heated to the preset melting temperature of the materials, the printing filaments were extruded. During the extrusion process, the deposition lines were arranged in an orderly fashion on the printing platform and were superimposed to form the work piece layer by layer. In the current study, the specimen was printed from a nozzle with an extrusion temperature of 250 °C onto a plate preheated to 100 °C with a printing speed of 60 mm/min, as suggested by the supplier. The rectangular specimens with dimensions of 9.5 mm × 80.0 mm × 4.2 mm were printed with different raster orientations and build directions, as shown in parts b and c of Figure 1, respectively. Each specimen had 42 layers with a controlled layer thickness of 0.1 mm.

### 2.2. Characterization

Thermogravimetric analysis (TGA) in the current study was performed using a Mettler TGA/DSC 3+ simultaneous thermal analyzer (Mettler Toledo Co., Greifensee, Switzerland). Specimens with a mass of 6.8 ± 2.1 mg were cut from the raw filament tows. The materials were heated from room temperature to 900 °C at a heating rate of 10 °C/min in a pure nitrogen atmosphere. The evolution of the weight of the materials as a function of temperature was recorded using the STARe software.

The microstructure of the cryofractured filament tows and the fractured printed composites was studied using scanning electron microscopy (Hitachi S-4800 FE-SEM, Hitachi, Japan). The morphology study focused on the adhesion states, feature dimensions, distribution, and dispersion of the short-carbon fibers and/or Kevlar fibers in the filament tows and printed components. 

Three-point bending tests were performed using an MTS universal mechanical testing machine (MTS E44, Eden Prairie, MN, USA) with a 30 kN loading cell. The bending tests were conducted at room temperature with a constant cross-head speed of 2.1 mm/min and according to ISO14125. The deflection of the samples during the mechanical tests was measured by a digital micrometer (Mitutoyo, ID-SX, Kawasaki, Japan). The flexure moduli of the materials were calculated from the initial slope of stress–strain curves over a strain range of 0.05–0.25%. The maximum stress was taken as the flexural strength. The flexural toughness of the materials was calculated by integrating the area under the stress–strain curve. Each measurement was averaged from the results of five repeated and recorded tests, in order to investigate the effects of fiber addition, raster orientations, and building directions on the flexure behaviors of ABS/CF/KF composites.

## 3. Results and Discussion

### 3.1. Thermal and Morphological Behaviors of the Filament Tows

**Figure 2:**
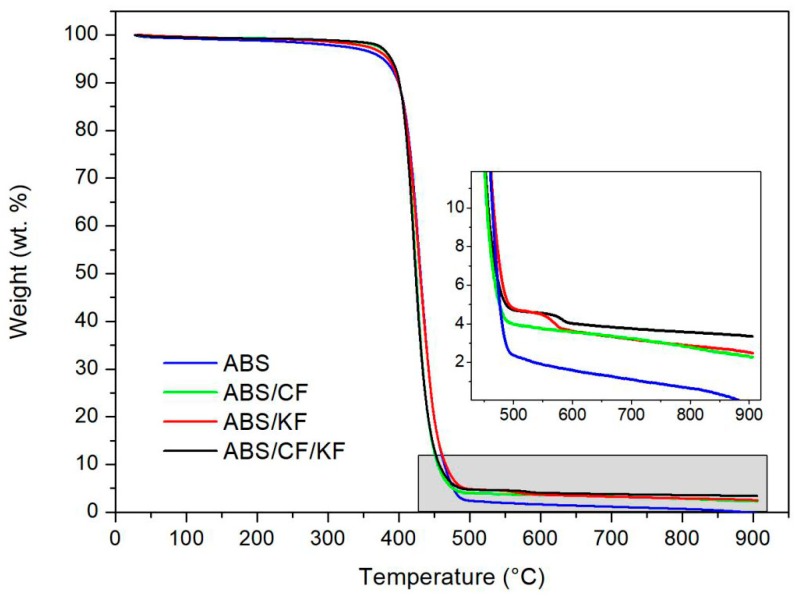
Thermogravimetric curves of pristine ABS, ABS/CF, ABS/KF, and ABS/CF/KF composites from room temperature to 900 °C under a nitrogen atmosphere.

TGA curves of the pristine ABS, ABS/CF, ABS/KF, and ABS/CF/KF composites are presented in Figure 2. In this figure, ABS and the ABS/CF composite show only one mass loss step in the temperature range of 300–500 °C. However, the degradation process of the ABS/KF and ABS/CF/KF composites is a two-step process with a substantial weight loss in the range from 350–500 °C and a second weight loss in the range from 500–600 °C. In addition, the initiated decomposition temperatures of the ABS/CF, ABS/KF, and ABS/CF/KF composites are slightly higher than that of pristine ABS in the range of 300–400 °C. In the temperature range from 400–500 °C, the ABS/KF composite and pristine ABS exhibit similar decomposition behaviors. However, the addition of CFs accelerates the degradation processes of the ABS/CF and ABS/CF/KF composites, resulting in an elevated slope of the weight loss. Upon increasing the temperature to 900 °C, ABS degrades completely and other materials retain carbon residues.

The one weight-loss step that occurs for ABS and the ABS/CF composite in Figure 2 could be attributed to the radical degradation process in ABS under an inert atmosphere [26]. The second degradation process of the ABS/KF and ABS/CF/KF composites is due to the decomposition of the Kevlar main chain [27]. The introduction of CFs could improve the thermal stability of the CF, ABS/CF, and ABS/CF/KF composites due to a higher degradation temperature than those of ABS and the ABS/KF composite [28]. On the other hand, the addition of CFs also improves the thermal conductivity of the ABS/CF and ABS/CF/KF composites, causing steeper weight reduction slopes than those for ABS and the ABS/KF composite [29]. Finally, the residues at the end of the heating process can be used to prove the presence of the reinforcements. Taking the TGA curve of ABS as a baseline, the CF and KF contents in the ABS/CF and ABS/KF composites are 2.48 wt % and 4.42 wt %, respectively. Also, the CF and KF contents in the ABS/CF/KF composites are 3.34 wt % and 3.05 wt %, respectively.

**Figure 3:**
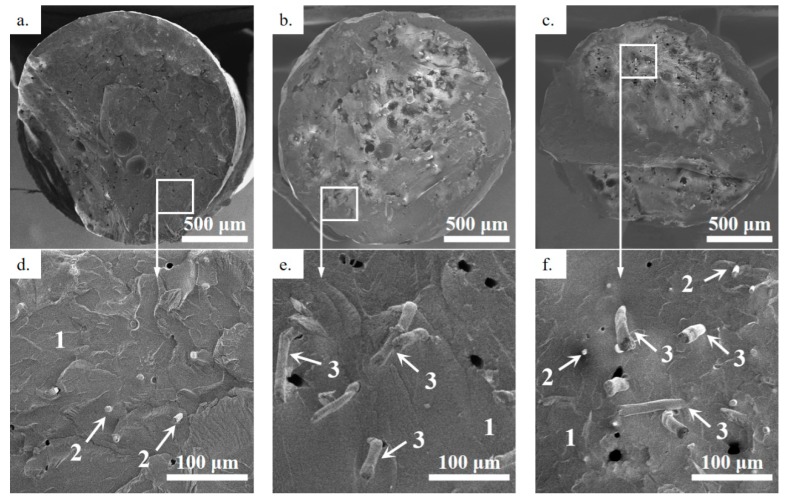
SEM images of cryofractured cross-sections of (**a**,**d**) ABS/CF, (**b**,**e**) ABS/KF, and (**c**,**f**) ABS/CF/KF tows, where arrows 1, 2, and 3 indicate the ABS matrix, short CFs, and short KFs, respectively.

Scanning electron microscopy (SEM) was used to investigate the cryofractured morphology of pristine ABS and the ABS/CF, ABS/KF, and ABS/CF/KF composite filaments employed in this study. As shown in Figure 3, both the CFs and KFs are well dispersed and distributed within the ABS matrix (arrow 1). In Figure 3d,f, the small grayish cylinders, indicated by arrow 2, indicate the short CFs with an average diameter of 5.8 ± 0.5 μm oriented along the filament length [30]. In Figure 3e,f, the large stick-like fillers, indicated by arrow 3, are KFs. Note that the diameter of the short KFs is 12 ± 0.6 μm, twice that of the CFs. The fibers that were pulled out during the cryofracture process lead to some voids (black dots) in the ABS matrix. Most of the fibers demonstrate a good interface with the ABS matrix. 

### 3.2. Flexural Behavior of the Printed Materials 

#### 3.2.1. Effect of Raster Orientations

**Figure 4:**
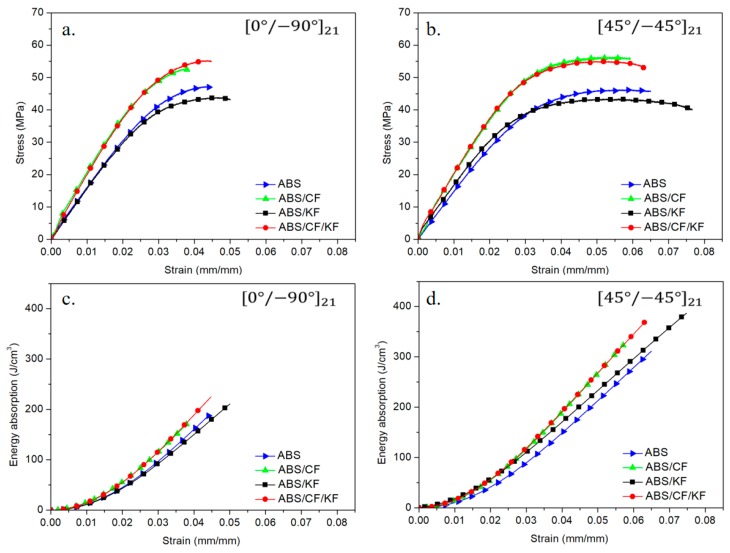
Stress–strain and energy absorption–strain curves of ABS, ABS/CF, ABS/KF, and ABS/CF/KF samples (**a**,**c**) printed at 0°/90° and (**b**,**d**) printed at ±45°.

The flexural behaviors of pristine ABS and the composites of ABS/CF, ABS/KF, and ABS/CF/KF with different raster orientations are shown in Figure 4. In Figure 4a,b, the stress–strain curves of all the studied materials show a linear initial stage. At the end of this linear stage, the stress–strain curves display a nonlinear transition to a yielding state and then a viscoplastic state, followed by the rupture of the materials. The extracted flexural moduli for the composites are summarized in Table 2. These results show that the flexural strengths and moduli of the composites increase substantially with the addition of a small amount of short CFs, regardless of the raster orientation (Table 2). Specifically, in Figure 4b, the flexural strength and modulus increase from 48.38 ± 1.80 MPa and 1419.52 ± 30.38 MPa for pristine ABS to 56.44 ± 1.60 MPa and 2253.48 ± 35.04 MPa for ABS/CF, representing increases of 16.7% and 58.7%, respectively. However, the ABS/CF composite exhibits lower elongation than pristine ABS for both the raster orientations in our investigated range (Figure 4a,b). With the reinforcement of the KFs, the ductility of the ABS/KF composite shows a significant improvement for both the cases with different raster orientations. However, the addition of the KFs obviously decreases the flexure strength of ABS. In the ABS/CF/KF composite, the decrease in the strength induced by the addition of the KFs could be compensated by the reinforcement of the CFs, resulting in a simultaneous enhancement of both the rigidity and ductility. The stress–strain curves for the materials with raster orientations of 0°/90° show lower ductility than those for the composites with raster orientations of ±45°. In Figure 4c,d, the energy absorption properties of the materials as a function of strain show that the composites with raster orientations of ±45° present higher energy absorption capabilities than the materials with raster orientations of 0°/90° at a given strain. The ABS/CF/KF composite show the highest flexural toughness obtained herein for both the cases with different raster orientations.

**Figure 5:**
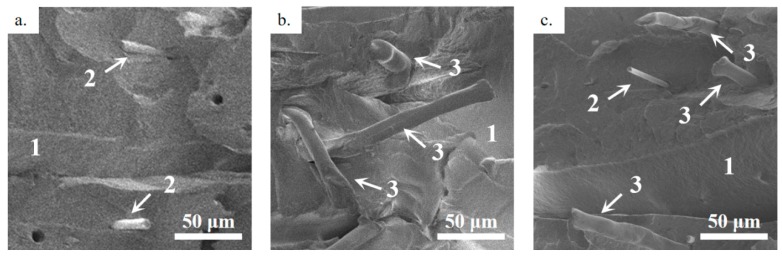
SEM images of (**a**) ABS/CF, (**b**) ABS/KF, and (**c**) ABS/CF/KF samples printed at ±45° after the three-point bending test.

SEM images of the fractured surfaces of the ABS-based composites with raster orientations of ±45° after flexural tests are shown in Figure 5. Other SEM images for the fractured composites with raster orientations of 0°/90° are not shown here because they led to similar conclusions. In Figure 5, it can be observed that short CFs and KFs are aligned with the line deposition direction. Compared to pristine ABS, the increased strength and modulus of the ABS/CF composite could be attributed to the addition of increasingly rigid CFs as well as the good interfaces between fillers and matrix [31]. The decreased ductility of the ABS/CF composite could be explained by the brittle CFs, which demonstrate smooth fractured CF surfaces with little plastic deformation, while the fractured KF surfaces show dimples that are characteristic of microvoid coalescence with a large amount of plastic deformation [19,32]. As shown in Figure 5b,c, the short KFs present relatively rough wavy fractured surfaces with microvoids, suggesting a large amount of plastic deformation. Such phenomenon could be linked to the toughening effects of short KFs in the composites of ABS/KF and ABS/CF/KF [33,34]. As the ABS/CF/KF composite in the current study demonstrated a good balance between rigidity and ductility, as shown in Figure 4, the following study focuses on ABS/CF/KF composites. In the following sections, we analyze the cross-sectional graphs of both undeformed and deformed samples of the ABS/CF/KF composite built with raster orientations of 0°/90° and ±45°.

**Figure 6:**
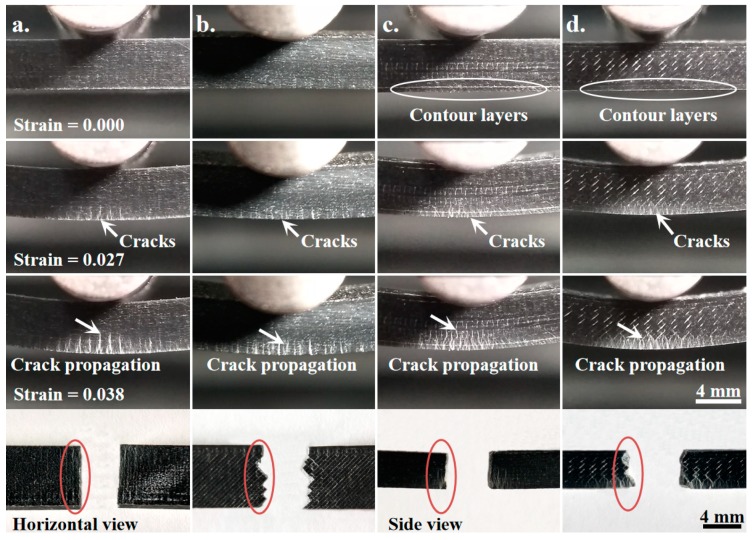
Cross-sectional graphs of the ABS/CF/KF composites show the details of the failure modes for the different raster orientations and build directions with (**a**) 0°/90° horizontal build, (**b**) ±45° horizontal build, (**c**) 0°/90° side build, and (**d**) ±45° side build.

**Figure 7:**
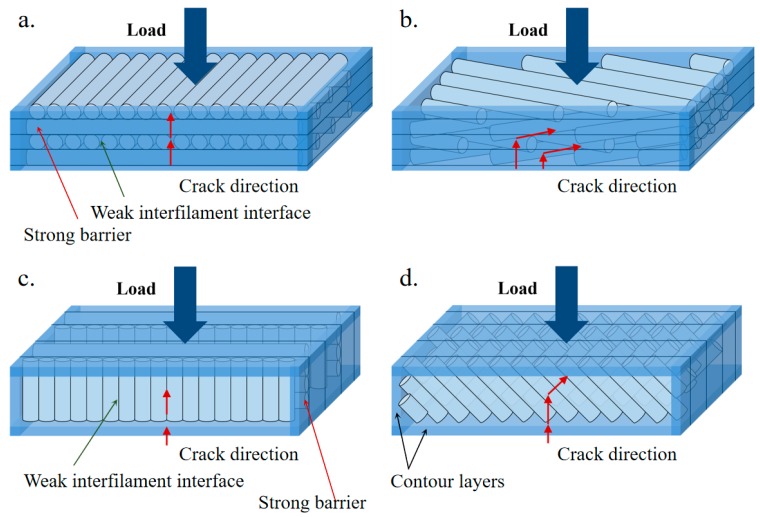
Schematic representation of crack propagation of the ABS/CF/KF composites with (**a**) 0°/90° horizontal build, (**b**) ±45° horizontal build, (**c**) 0°/90° side build, and (**d**) ±45° side build.

The crack initiation and propagation in the composite of ABS/CF/KF with different raster orientations and build directions during the bending test are illustrated in Figure 6. With increasing strain up to 0.027, microcracking initiates and white cracks appear at the bottom for all specimens. At a strain of 0.038, cracks eventually climb along the loading directions and additional cracks are created. Specifically, after crack initiation, the cracks of the composites with a ±45° orientation propagate along the initial crack direction with a shorter distance than those of the composites with a 0°/90° orientation at a given strain. Finally, the composites of ABS/CF/KF with a raster orientation of 0°/90° fracture with a planar cross section, while those with a raster orientation of ±45° exhibit a very jagged fractured surface. The differences in the crack propagation behavior of the composites may be due to the different relationships between the layers and crack planes in the present work. As schematically presented in Figure 7a, in the case of a raster orientation of 0°/90°, the 0° layers acting as strong barriers are pulled under tension along the filaments that are perpendicular to the propagation plane, delaying crack propagation [35]. However, the 90° layers are parallel to the propagation plane. The breakage of interfilaments creates paths that facilitate the propagation of cracks [36]. Therefore, only half of the filaments for the composites could resist crack propagation, resulting in a relatively low fracture toughness [37]. In the case of a raster orientation of ±45°, all filaments have an angle of 45° against the loading direction. The filaments act as strong barriers that retard the crack propagation along the weak interfilament interfaces. The cracks propagate through irregular zigzag paths, increasing the fracture toughness by dissipating the fracture energy over a larger volume (Figure 7b).

#### 3.2.2. Effect of Building Directions

**Figure 8:**
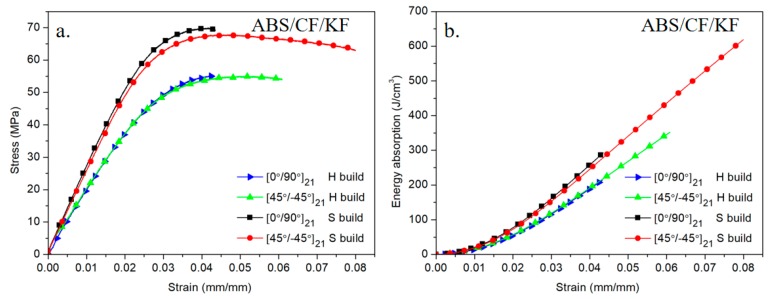
(**a**) Stress-strain curves and (**b**) energy absorption-strain curves of ABS/CF/KF composites with different building directions.

The flexural behaviors of the ABS/CF/KF composites with different build directions and raster orientations are shown in Figure 8a. The stress–strain curves for the composites with a side build direction show higher initial linear slopes than those for the composites with a horizontal build direction. As shown in Table 3, moduli of the ABS/CF/KF composites horizontally printed at 0°/90° and ±45° increased from 2167.17 ± 36.69 MPa and 2110.08 ± 29.79 MPa to 2683.33 ± 36.17 MPa and 2513.33 ± 43.12 MPa for the composites with a side-build direction, representing increases of 23.8% and 19.1%, respectively. In addition, the flexural strengths of composites with a side build direction showed a significant improvement in both the raster orientations in our studied ranges. The ultimate flexural strain of the composite with a side build direction and a raster orientation of ±45° is much higher than that of the composite with a horizontal build direction and with the same raster orientation. (Figure 8 (a)). In Figure 8 (b), the energy absorption properties of the materials as a function of strain show that the composites with a side build direction presented higher energy absorption capabilities than those for the materials with a horizontal direction at a given strain. The composites with a raster orientation of ±45° and side build direction showed the highest energy absorption capacity obtained in this work.

As shown in Figure 6, the deformation phenomena of the composites with side build directions are different from those of composites with horizontal build directions. Specifically, even though the microcracks initiated at the bottom for all composites, they formed on the deposited layers for composites in a horizontal build direction rather than on the contour layers for composites with a side build direction. The multiple contour layers constructed with a raster orientation of 0° might offer an elevated resistance to bending due to the effective long raster lengths, resulting in elevated flexure moduli and strengths [38]. The raster direction also plays an important role in the crack propagation rate. The side build composites stretch to a strain of 0.038, the cracks extend to 1.82 ± 0.09 mm for the composites printed with the 0°/90° raster orientation and only extend to 1.12 ± 0.05 mm for the composites printed with the ±45° raster orientation, showing a different crack propagation velocity. The crack propagation of the composites with the ±45° raster orientation show a zigzag path, but the composite with the 0°/90° raster orientation exhibits a flat crack path. Similar to the composites with a horizontal build direction, the elevated fracture toughness for the composites with a raster orientation of ±45° can be attributed to the zigzag path for crack propagation (Figure 7c,d).

## 4. Conclusions

In this work, we analyzed the mechanical and thermal properties of 3D-printed ABS-based composite specimens with different reinforcements (CFs and/or KFs). The samples were manufactured via a material extrusion 3D-printing technique using two different raster orientations and different build directions. The morphological structures at different tensile strains were investigated by digital camera and scanning electron microscopy (SEM). It was found that both CFs and KFs were well dispersed and distributed within the ABS matrix. Thermal testing showed that the introduction of CFs improved both the thermal conductivity and stability of the composites. The mechanical testing results revealed that the addition of short CFs increased the flexure modulus and strength, but decreased the ultimate flexure strain of the composites. However, the introduction of KFs increased the ductility of 3D-printed ABS/KF specimens because of the toughening effect induced by the short KFs. In addition, the ABS-based composites containing both carbon and Kevlar fibers simultaneously showed improved rigidity and ductility, which is suitable for energy absorption applications. Moreover, the deformation and failure modes of the composite specimens printed with different raster orientations indicated that the irregular zigzag crack propagation paths for the composites printed with a raster orientation of ±45° delayed the breakage of the materials. The contour of the printed composites further postponed the initiation and propagation of cracks. The ABS/CF/KF composite specimens printed at a ±45° raster orientation with a side build direction had the highest flexure toughness with an improved strength and ductility among all specimens investigated in this study.

## Figures and Tables

**Table 1:** Properties of carbon and Kevlar fibers within filaments.

Fiber	Mean Length (μm)	Mono Fiber Diameter (μm)	Fiber Population (Unit/g of Filament)
CF	251.00	7.00 ± 2.00	4.37 × 10^6^
KF	215.00	10.00 ± 2.00	5.00 × 10^6^

**Table 2:** Flexural moduli of ABS, ABS/CF, ABS/KF, and ABS/CF/KF composites.

Material	Raster Orientation	Flexural Strength (MPa)	Flexural Modulus (MPa)
ABS	0°/90°	48.45 ± 1.55	1563.00 ± 44.10
	±45°	48.38 ± 1.80	1419.52 ± 30.38
ABS/CF	0°/90°	53.73 ± 1.35	2351.00 ± 31.51
	±45°	56.44 ± 1.60	2253.48 ± 35.04
ABS/KF	0°/90°	45.50 ± 0.66	1618.67 ± 39.22
	±45°	43.40 ± 1.35	1730.78 ± 47.31
ABS/CF/KF	0°/90°	56.46 ± 0.55	2167.17 ± 36.69
	±45°	55.51 ± 2.19	2110.08 ± 29.79

**Table 3:** Flexural behaviors of ABS/CF/KF composites with different raster orientations and building directions.

Raster Orientation	Building Direction	Flexural Strength (MPa)	Flexural Modulus (MPa)
0°/90°	Horizontal	56.46 ± 0.55	2167.17 ± 36.69
	Side	69.87 ± 0.24	2683.33 ± 36.17
±45°	Horizontal	55.51 ± 2.19	2110.08 ± 29.79
	Side	67.92 ± 0.09	2513.33 ± 43.12
